# Supplementary material for: Structural basis of epitope selectivity and potent protection from malaria by PfCSP antibody L9
Source: Nat Commun. 2023 May 17;14:2815. doi: 10.1038/s41467-023-38509-2 (PMC10192352; doi:10.1038/s41467-023-38509-2)
Supplement: Supplementary file 1 — Supplementary Information [file 41467_2023_38509_MOESM1_ESM.pdf]

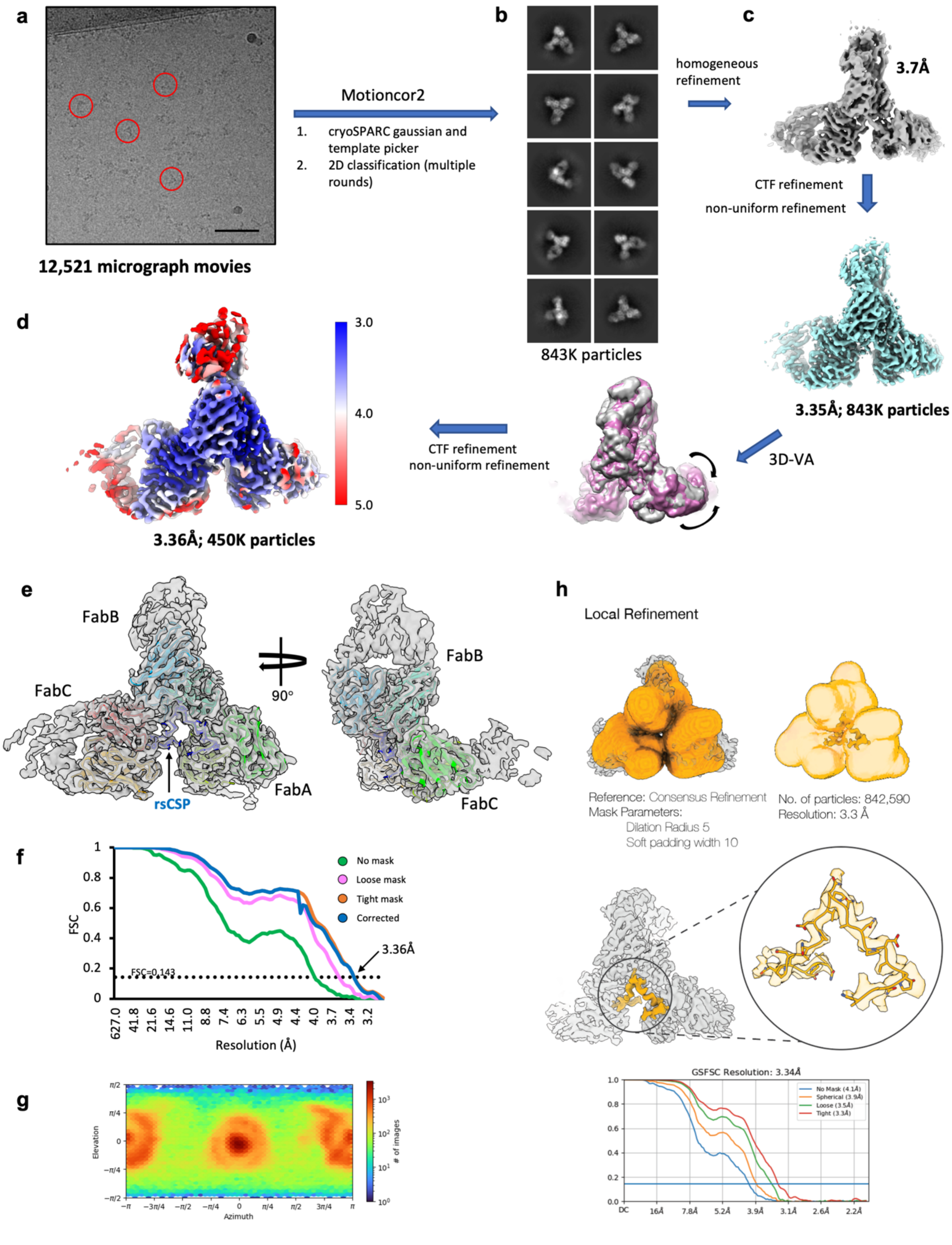
a
12,521 micrograph movies
Motioncor2
1. cryoSPARC gaussian and template picker
2. 2D classification (multiple rounds)
b
843K particles
homogeneous refinement
c
3.7Å
CTF refinement
non-uniform refinement
3.35Å; 843K particles
3D-VA
d
CTF refinement
non-uniform refinement
3.0
4.0
5.0
3.36Å; 450K particles
e
FabB
FabC
FabA
rsCSP
90°
h
Local Refinement
Reference: Consensus Refinement
Mask Parameters:
Dilation Radius 5
Soft padding width 10
No. of particles: 842,590
Resolution: 3.3 Å
f
FSC
No mask
Loose mask
Tight mask
Corrected
FSC=0.143
3.36Å
Resolution (Å)
g
Elevation
Azimuth
# of images
GSFSC Resolution: 3.34Å

Supplementary Figure 1. **Cryo-EM reconstruction of L9-rsCSP and data processing workflow.** **a** Representative cryo-EM micrograph of L9 on graphene oxide. Scale bar = 50 nm. Cryo-EM datasets were collected across 2 protein preparations and four data collection sessions. Initially each dataset was processed independently, and the reconstructions yielded identical structures. Thus, for the final reconstructions (d and g), particles across all datasets were pooled and processed together. See Methods. **b** Representative 2D class averages. **c** Intermediate cryo-EM maps. Top: initial consensus refinement; Middle: high-resolution map with full data set; Lower left: representative cluster maps from 3D variability analysis (VA) of full dataset in CryoSPARC v3.3[1], showing motion in Fab A. CTF: contrast transfer function. **d** Local resolution map of final consensus reconstruction after 3D-VA and further CTF refinement. **e** Overlay of final L9-rsCSP structure with cryo-EM map in d. **f** Angular distribution plot of map in d. **g** Fourier shell correlation (FSC) plot of map in d. **h** Local refinement in CryoSPARC v4.0, using full particle dataset, to improve quality of L9 variable (Fv) and PfCSP reconstructions. The mask used is shown in the top two panels in orange.

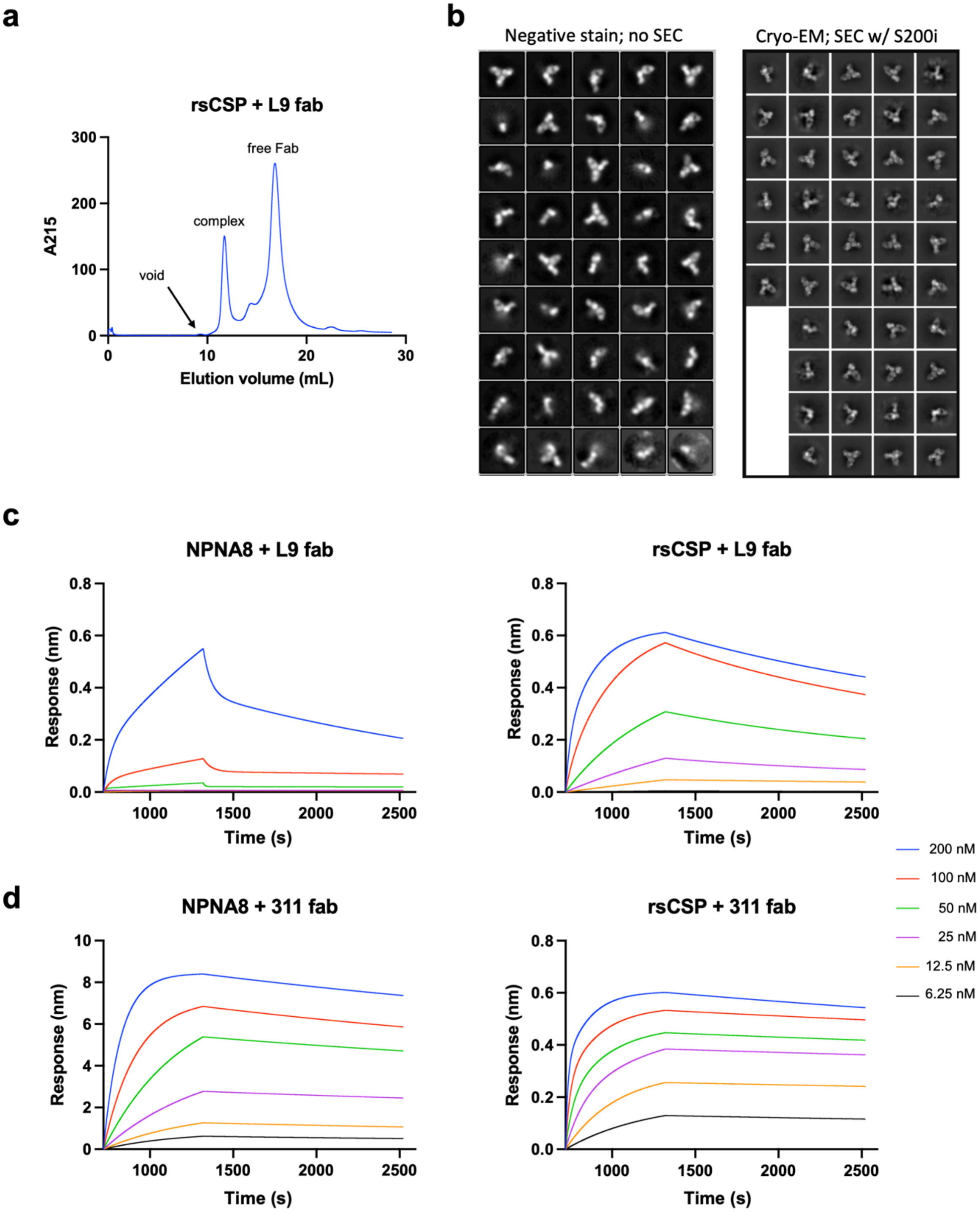

e

mAb	NPNA$_8$ K_D (nM)	NPNA$_8$ K_{on} (1/M*s)	NPNA$_8$ K_{dis} (1/s)	rsCSP K_D (M)	rsCSP K_{on} (1/M*s)	rsCSP K_{dis} (1/s)
L9	6260	3.2E4	2.4E-2	14	5.5E4	4.5E-4
311	2.2	1.1E5	1.5E-4	0.3	2.4E5	7E-5

Supplementary Figure 2. **Biophysical characterization of L9 Fab.** **a** Size exclusion (SEC) profile of L9 Fab with rsCSP using a Superdex200 Increase 10/300 column. The fractions encompassing the peak labelled "complex" were pooled and used for cryo-EM studies. **b** Observed 2D class averages of L9-rsCSP complexes with either negative stain EM, without SEC purification, or cryo-EM, which resulted from the "complex" peak in a. **c** Biolayer interferometry (BLI) data L9 binding to $NPNA_8$ peptide (left), or rsCSP. The fits to the binding response data are shown, and were calculated using a 2:1 kinetic binding model in the Octet Data Analysis software. In all experiments, CSP peptides were immobilized to Streptavidin (SA) biosensors, and the concentration of Fab in solution was varied, as shown in legend. **d** Same as in c, for 311 Fab, which has very high avidity to the major NPNA repeats. **e** Calculated kinetic parameters for L9 and 311. Parameters were computed as an average of at least four concentrations of Fab with $R^2 \geq 0.95$**.** Source data are provided as a Source Data file.

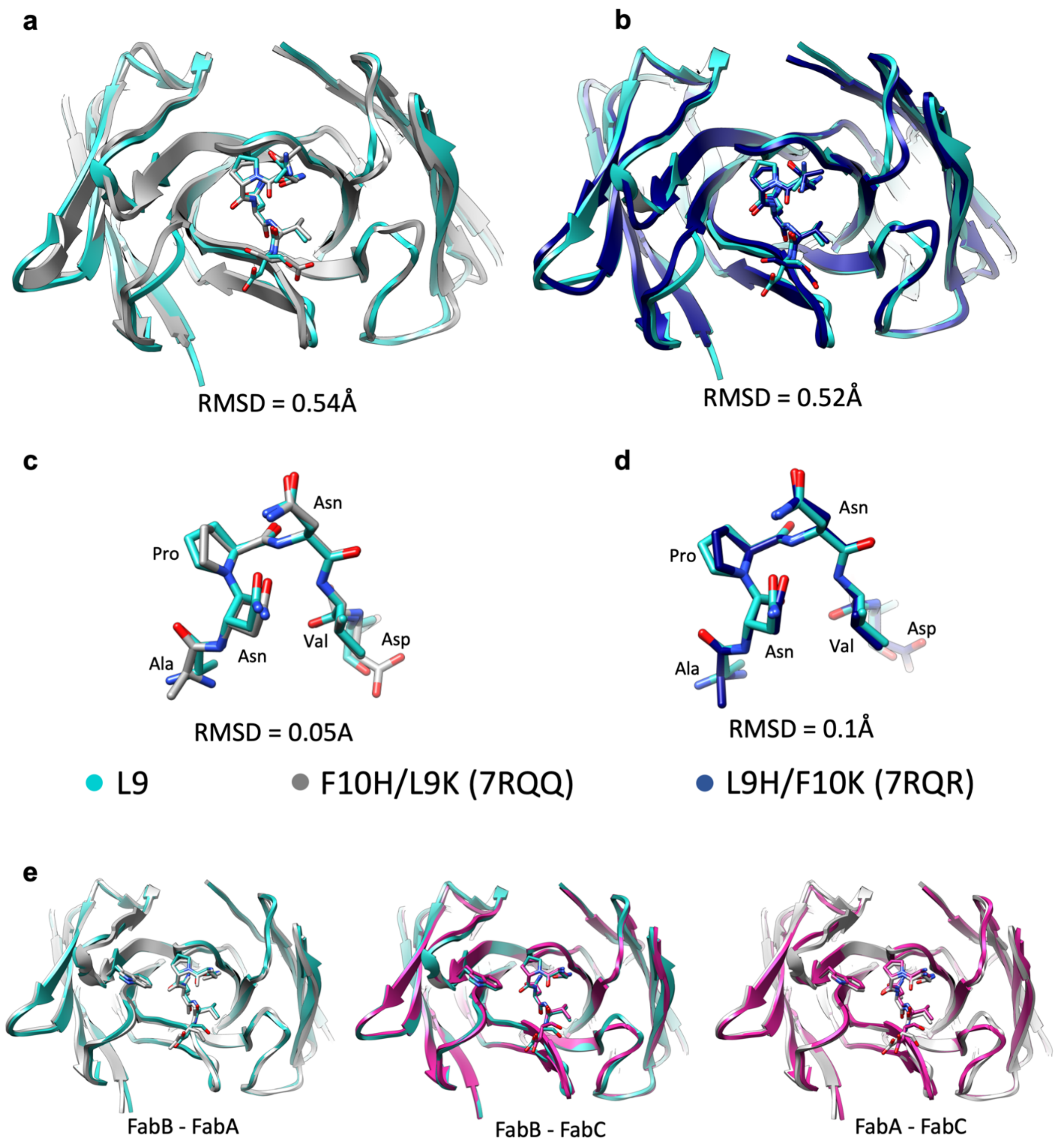

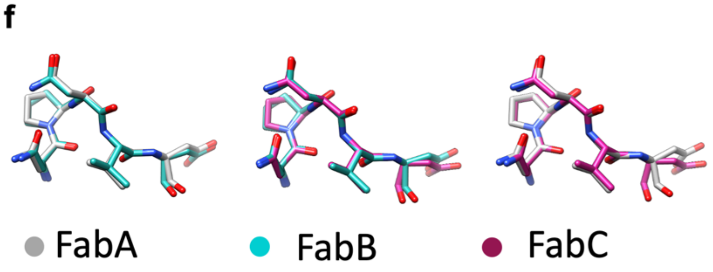

g

Comparison	Fv RMSD	Epitope RMSD
FabB - FabA	0.43Å	0.11Å
FabB - FabC	0.43Å	0.07Å
FabA - FabC	0.5Å	0.05Å

Supplementary Figure 3. **Structural comparison of L9 Fab cryo-EM structure with X-ray structures of L9 chimeras. a** Overlay of single L9 Fab and rsCSP (ANPNVD) with $F10_H/L9_K$ bound to NANPNVD, where NPNV forms a type 1 β-turn. RMSD: root mean square deviation. **b** Structural match of PfCSP peptides from L9 cryo-EM and $F10_H/L9_K$ X-ray structures. **c,d** Same as in a and b, for $L9_H/F10_K$. All RMSD values are computed for Cα atoms in UCSF Chimera[2]. **e** Structural match of the three Fab copies in the L9 cryo-EM structure. The three Fabs are highly similar, with no significant variations observed. **f** Same as in e, aligning only the primary NPNVD PfCSP epitope from each Fab. **g** Computed Cα RMSD values for structural alignments in e and f. Calculations performed in UCSF Chimera.

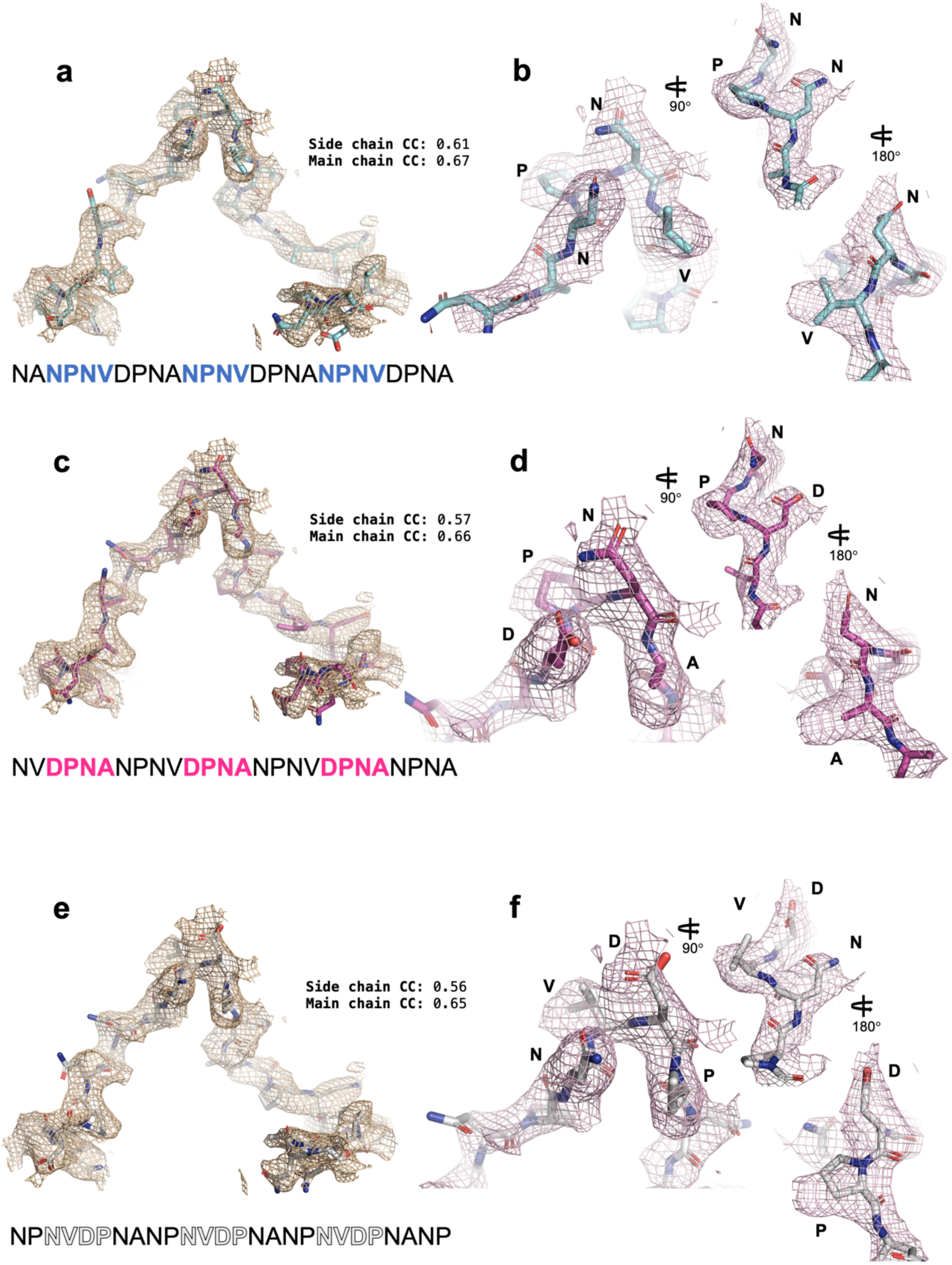
a
Side chain CC: 0.61
Main chain CC: 0.67
NANPNVDPNANPNVDPNANPNVDPNA
b
90°
180°
c
Side chain CC: 0.57
Main chain CC: 0.66
NVDPNANPNVDPNANPNVDPNANPNA
d
90°
180°
e
Side chain CC: 0.56
Main chain CC: 0.65
NPNVDPNANPNVDPNANPNVDPNANP
f
90°
180°

Supplementary Figure 4. **Quality of fit of original and potential alternate registrations of minor repeat region of rsCSP.** **a** Structure of rsCSP minor repeats as originally modelled; cryo-EM density is shown as mesh and displayed at 3σ. Side chain and main chain cross-correlation (cc) coefficients to the map are listed. The sequence of the modelled peptide is shown, with the core L9-interacting repeats (NPNV) in blue. **b** Zoomed in view of central NPNV repeat; cryo-EM density map is displayed at 4σ. **c,d** Same as in a and b, with the registration of rsCSP shifted by one repeat, making the core interacting repeat DPNA. Alternate structures were first calculated in Coot[3], and then refined into the map with L9 with PHENIX[4]. This model is unlikely given the poor quality of fit and prior structural and functional data showing that L9 binds NPNV[5,6]. **e,f** Same as in a and b, with the registration altered by two residues such that the minor repeat Asp assumes the role of N3 to engage $R96^{L}$ in CDRL3, yielding NVDP as the core L9-interacting repeat. This model is unlikely for the same reasons listed in c and d, and produces a type-1 beta turn within NVDP, which has not been observed.

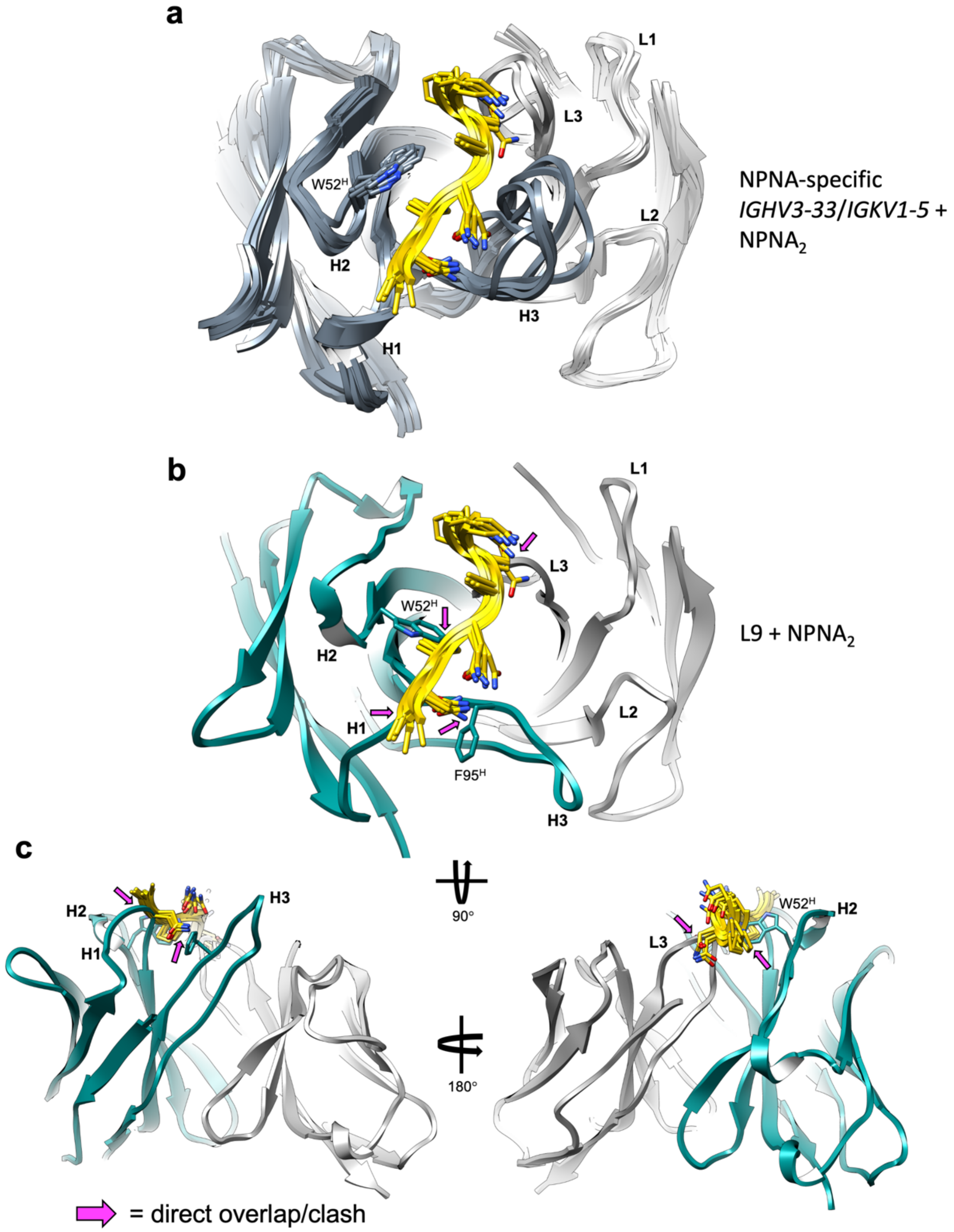
a
L1
L3
W52H
L2
H2
H3
H1
NPNA-specific
IGHV3-33/IGKV1-5 +
NPNA2
b
L1
L3
W52H
H2
L2
H1
F95H
H3
L9 + NPNA2
c
H2
H3
H1
90°
180°
W52H
H2
L3
= direct overlap/clash

Supplementary Figure 5. **Structure of L9 paratope disfavors $NPNA_2$ binding. a** Superposition of NPNA-specific IGHV-33/IGKV1-5 Fabs. mAbs shown are the same as in Figure S3G; L9 not shown. All structures are aligned to a single L9 Fab. Germline $W52^H$ side chain is shown for reference. Heavy and light chain CDR loops are specified as H1, L1, etc. **b** Same as in a, showing only L9 Fv and $NPNA_2$ structures from a. Pink arrow denotes direct clash of $NPNA_2$ peptide with L9 Fv, suggesting that the L9 paratope conformation is not competent to bind NPNA peptides. **c** Same as in b, rotated 90 degrees to show direct clash with CDRH2, CDRH3 (left) and CDRL3 (right) loops.

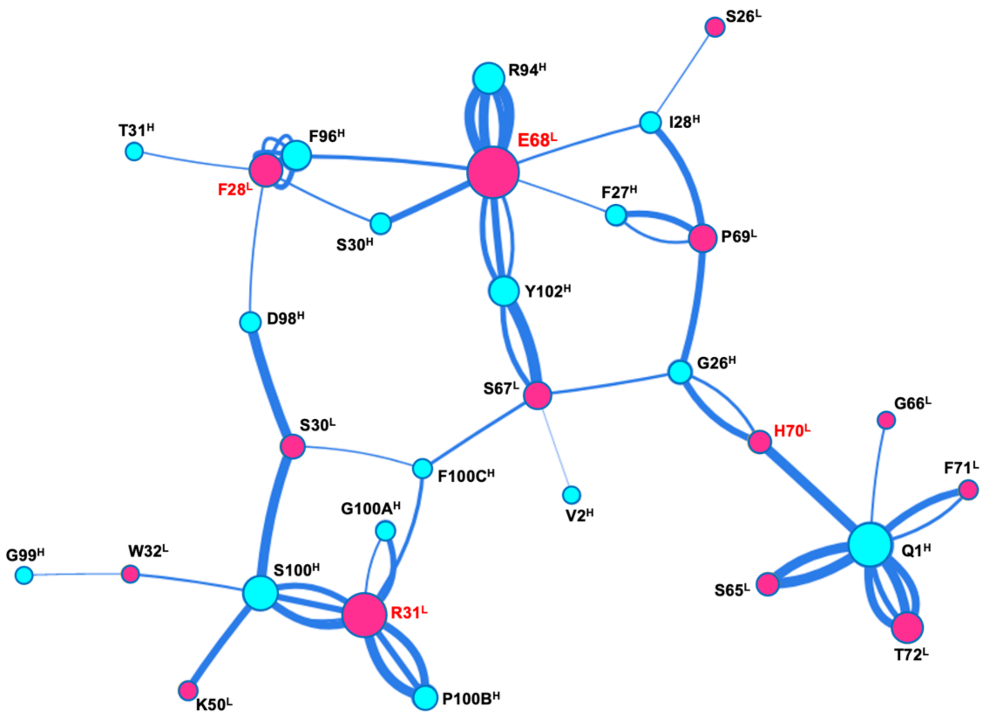

Supplementary Figure 6. **Contact network of the L9 homotypic interface.** Light chain residues are colored in magenta; heavy chain residues are in cyan. The size of each circle corresponds to the overall contribution of that residue to the homotypic interface. The width of the lines indicates the strength of the homotypic contact. Contact network generated with the Epitope Analyzer webtool[7].

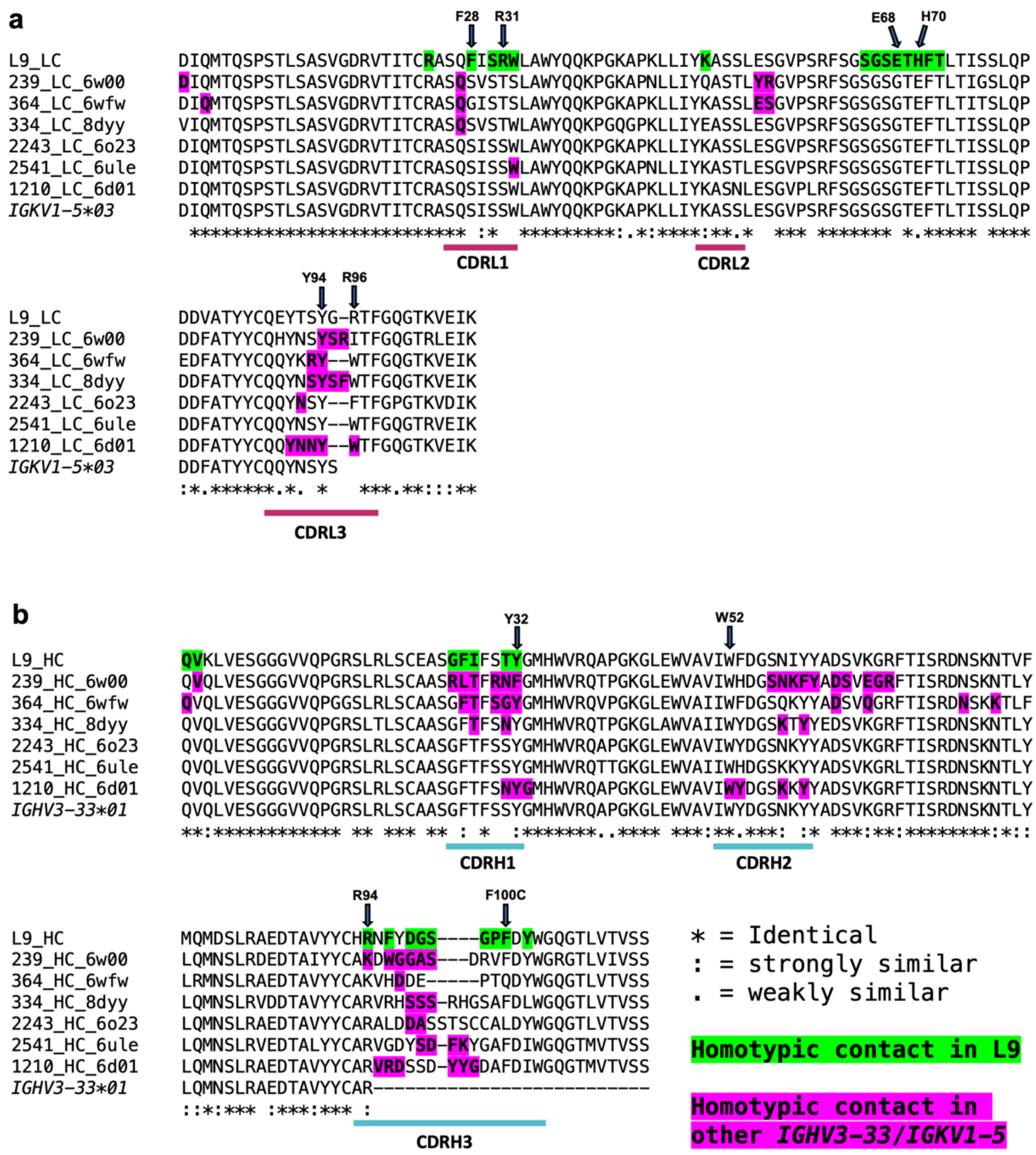

Supplementary Figure 7. **Multiple sequence alignment (MSA) of L9 with NPNA-targeting mAbs.** All mAbs utilize the same light and heavy chain germline genes, *IGKV1-5* and *IGHV3-33*, respectively. PDB codes follow mAb names. **a** Light chain MSA. **b** Heavy chain MSA.

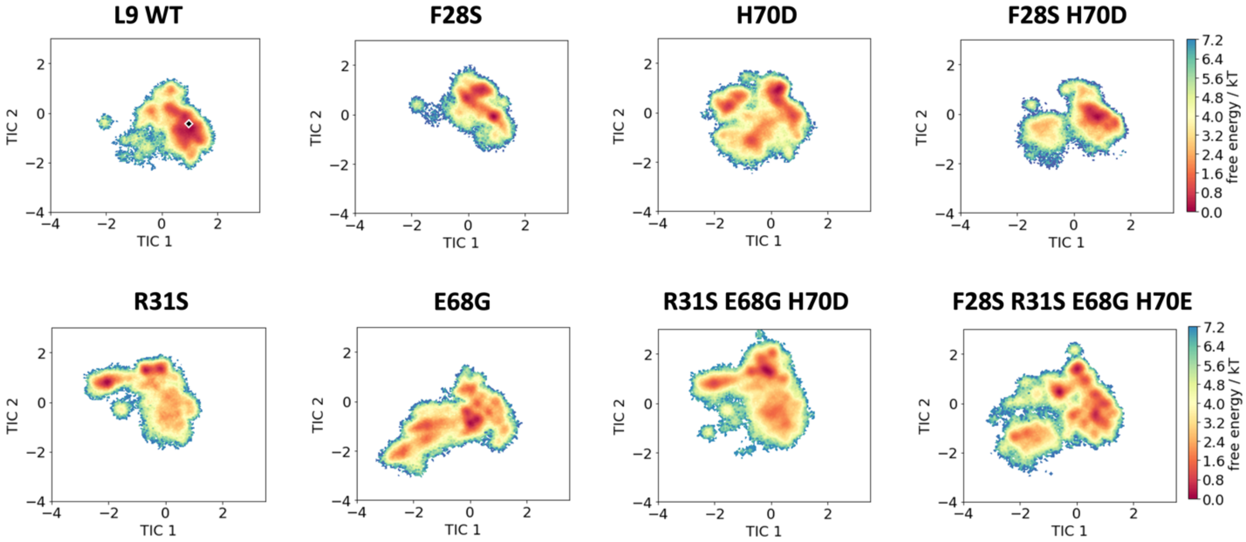

Supplementary Figure 8**. Free energy landscapes of isolated Fv domains of all variants modelled.** See also Figure 5. *k*: Boltzmann constant; T: temperature.

Table S1. Cryo-EM data collection parameters and model statistics

	L9 Fab-rsCSP<br>PDB: 8EH5<br>EMDB: 28135
**Data Collection**	
Microscope	Titan Krios
Detector	Gatan K2 Summit
Voltage (kV)	300
Pixel Size (Å)	1.045
Defocus range (μm)	-0.9 to -2.0
Total electron dose ($e^-/Å^2$)	60
Dose rate ($e^-/Å^2$/sec)	6
Frames per exposure	50
**Data Processing**	
Total micrograph movies	12,521
Particle images in map	451,712
Symmetry imposed	C1
Map resolution (FSC=0.143; Å)	3.36
Map sharpening B-factor ($Å^2$)	136.9
Data processing software	cryoSPARC v3.3
**Model Refinement**	
No. atoms in deposited model	5509
Chains total	7
Residues (protein)	704
RMS Deviations	
Bond lengths (Å)	0.022
Bond angles (°)	1.719
Validation	
MolProbity score	1.37
Clash score	2.69
EMRinger score	3.22
Poor rotamers (%)	0.5
Ramachandran plot	
Favored (%)	95.5
Allowed (%)	3.9
Outliers (%)	0.6
Average B-factor	47

Table S2. Direct contacts between central L9 Fab and rsCSP

Chain 1	Residue 1	Position 1	Atom (1>2)	Chain 2	Residue 2	Position 2	Distance (Å)	Predicted interaction
G	ASN	115	ND2-CD2	L	TRP	32	3.2	Van-der-Waals
G	ASN	115	ND2-O	L	TYR	91	2.9	H-bond
G	ASN	115	ND2-CA	L	THR	92	3.7	Van-der-Waals
G	ASN	115	ND2-O	L	THR	92	3.9	H-bond
G	PRO	116	O-CE2	H	TYR	32	3.4	Van-der-Waals
G	PRO	116	O-CZ2	H	TRP	52	3.6	Van-der-Waals
G	PRO	116	CD-O	L	THR	92	3.9	Van-der-Waals
G	PRO	116	CD-CA	L	SER	93	4.3	Van-der-Waals
G	PRO	116	CB-CZ	L	TYR	94	3.6	Hydrophobic
G	PRO	116	CB-CE2	L	TYR	94	3.6	Hydrophobic
G	PRO	116	CG-CZ	L	TYR	94	3.7	Hydrophobic
G	PRO	116	CG-CE1	L	TYR	94	3.8	Hydrophobic
G	PRO	116	CB-CE1	L	TYR	94	4.1	Hydrophobic
G	PRO	116	CG-CE2	L	TYR	94	4.2	Hydrophobic
G	PRO	116	CB-CD2	L	TYR	94	4.3	Hydrophobic
G	PRO	116	CG-CD1	L	TYR	94	4.3	Hydrophobic
G	ASN	117	OD1-CH2	H	TRP	52	4.0	Van-der-Waals
G	ASN	117	OD1-O	H	ASN	95	4.1	Van-der-Waals
G	ASN	117	O-CA	H	PHE	96	3.4	Van-der-Waals
G	ASN	117	O-N	H	TYR	97	2.9	H-bond
G	ASN	117	ND2-O	L	TYR	91	3.5	H-bond
G	ASN	117	ND2-O	L	SER	93	2.9	H-bond
G	ASN	117	OD1-NH2	L	ARG	96	2.8	H-bond
G	ASN	117	OD1-NH1	L	ARG	96	2.9	H-bond
G	VAL	118	C-OH	H	TYR	32	4.1	Van-der-Waals
G	VAL	118	O-OH	H	TYR	32	4.4	H-bond
G	VAL	118	CA-O	H	TYR	97	3.5	Van-der-Waals
G	VAL	118	CG2-CB	H	TYR	97	4.2	Hydrophobic
G	VAL	118	CG2-CD2	H	TYR	97	4.3	Hydrophobic
G	VAL	118	N-O	H	TYR	97	4.4	H-bond
G	VAL	118	CG1-CB	H	TYR	97	4.4	Hydrophobic
G	VAL	118	CG2-CE1	L	TYR	91	4.3	Hydrophobic
G	ASP	119	CB-OH	H	TYR	32	3.4	Van-der-Waals
G	ASP	119	N-OH	H	TYR	32	3.6	H-bond
G	ASP	119	OD2-OH	H	TYR	32	4.4	H-bond
G	ASP	119	OD1-OH	H	TYR	32	4.4	H-bond
G	ASP	119	N-O	H	TYR	97	3.2	H-bond
G	ASP	119	CB-OD1	H	ASP	98	3.4	Van-der-Waals

Table S3. Homotypic interactions in L9-rsCSP cryo-EM structure (Fab B and Fab C)

Chain Fab 1	Residue 1	Position 1	Atom (1>2)	Chain Fab 2	Residue 2	Position 2	Distance (Å)	Predicted interaction
P (L)	SER	26	O-CD1	H	ILE	28	4.1	Van-der-Waals
P (L)	PHE	28	CB-OG	H	SER	30	3.8	Van-der-Waals
P (L)	PHE	28	CE1-OG1	H	THR	31	4.0	Van-der-Waals
P (L)	PHE	28	CZ-CE1	H	PHE	96	3.5	Hydrophobic
P (L)	PHE	28	CE1-CE1	H	PHE	96	3.5	Hydrophobic
P (L)	PHE	28	CE1-CZ	H	PHE	96	3.8	Hydrophobic
P (L)	PHE	28	CZ-CD1	H	PHE	96	4.2	Hydrophobic
P (L)	PHE	28	CZ-CZ	H	PHE	96	4.2	Hydrophobic
P (L)	PHE	28	CE1-CD1	H	PHE	96	4.5	Hydrophobic
P (L)	PHE	28	CE2-OD2	H	ASP	98	3.9	Van-der-Waals
P (L)	SER	30	OG-OD2	H	ASP	98	2.8	H-bond
P (L)	SER	30	O-OG	H	SER	100	2.8	H-bond
P (L)	SER	30	CA-CZ	H	PHE	100C	4.1	Van-der-Waals
P (L)	ARG	31	CD-OG	H	SER	100	3.6	Van-der-Waals
P (L)	ARG	31	NH1-O	H	SER	100	3.6	H-bond
P (L)	ARG	31	NH1-OG	H	SER	100	4.2	H-bond
P (L)	ARG	31	NE-OG	H	SER	100	4.3	H-bond
P (L)	ARG	31	NH1-CA	H	GLY	100A	3.8	Van-der-Waals
P (L)	ARG	31	NH1-O	H	GLY	100A	4.4	H-bond
P (L)	ARG	31	NH2-O	H	PRO	100B	2.8	H-bond
P (L)	ARG	31	NH1-O	H	PRO	100B	3.5	H-bond
P (L)	ARG	31	NH1-N	H	PRO	100B	4.1	H-bond
P (L)	ARG	31	NE-CE2	H	PHE	100C	3.3	Van-der-Waals
P (L)	TRP	32	CZ2-C	H	GLY	99	4.2	Van-der-Waals
P (L)	TRP	32	CZ2-N	H	SER	100	3.7	Van-der-Waals
P (L)	LYS	50	NZ-O	H	SER	100	3.8	H-bond
P (L)	SER	65	O-NE2	H	GLN	1	2.6	H-bond
P (L)	SER	65	OG-NE2	H	GLN	1	2.9	H-bond
P (L)	GLY	66	N-NE2	H	GLN	1	3.8	Van-der-Waals
P (L)	SER	67	CB-CG2	H	VAL	2	4.4	Van-der-Waals
P (L)	SER	67	O-O	H	GLY	26	3.5	Van-der-Waals
P (L)	SER	67	OG-CD2	H	PHE	100C	3.4	Van-der-Waals
P (L)	SER	67	OG-OH	H	TYR	102	2.2	H-bond
P (L)	SER	67	O-OH	H	TYR	102	4.5	H-bond
P (L)	GLU	68	OE1-CB	H	PHE	27	3.9	Van-der-Waals
P (L)	GLU	68	OE1-O	H	ILE	28	3.6	Van-der-Waals
P (L)	GLU	68	OE1-OG	H	SER	30	4.1	H-bond
P (L)	GLU	68	OE2-NE	H	ARG	94	2.8	H-bond
P (L)	GLU	68	OE2-NH2	H	ARG	94	3.6	Salt-Bridge
P (L)	GLU	68	OE1-NH2	H	ARG	94	3.8	Salt-Bridge
P (L)	GLU	68	OE1-NE	H	ARG	94	4.1	H-bond
P (L)	GLU	68	OE2-CZ	H	PHE	96	3.1	Van-der-Waals
P (L)	GLU	68	OE2-CE1	H	TYR	102	3.2	Van-der-Waals
P (L)	GLU	68	OE2-OH	H	TYR	102	3.9	H-bond
P (L)	GLU	68	N-OH	H	TYR	102	4.5	H-bond
P (L)	THR	69	OG1-O	H	GLY	26	3.9	H-bond
P (L)	THR	69	OG1-CA	H	PHE	27	3.5	Van-der-Waals
P (L)	THR	69	OG1-N	H	PHE	27	4.1	H-bond
P (L)	THR	69	OG1-N	H	ILE	28	3.8	H-bond
P (L)	HIS	70	ND1-OE1	H	GLN	1	2.8	H-bond
P (L)	HIS	70	CB-O	H	GLY	26	3.4	Van-der-Waals
P (L)	HIS	70	N-O	H	GLY	26	3.9	H-bond
P (L)	PHE	71	C-OE1	H	GLN	1	3.4	Van-der-Waals
P (L)	PHE	71	N-OE1	H	GLN	1	3.7	H-bond
P (L)	THR	72	OG1-OE1	H	GLN	1	2.9	H-bond
P (L)	THR	72	N-OE1	H	GLN	1	3.4	H-bond
P (L)	THR	72	OG1-NE2	H	GLN	1	3.6	H-bond
P (L)	THR	72	O-NE2	H	GLN	1	4.4	H-bond

Table S4. Computed interaction energies of homotypic interface in trimeric L9-rsCSP complexes

L9 Variant	Electrostatic Energy (kcal/mol)	SD	van der Waals Energy (kcal/mol)	SD	Sim. Time (μs)
L9	-150.2	32	-27.4	6	5
F28S*	-46.8	34	-9.5	5	5
F28S, H70D*	-82.6	28	-16.4	6	5
H70D*	-228.3	27	-41.4	8	5
E68G*	-39.3	44	-11.8	6	5
R31S	-170.4	30	-29.9	5	5
R31S, E68G, H70D*	-41.8	31	-14.7	8	5
F28S, R31S, E68G, H70E*	-18.9	33	-6.1	6	5
$F10_K$ $L9_H$ Chimera*	-20.2	32	-5.6	6	5
$L9_K$ $F10_H$ Chimera	-118.4	37	-12.3	7	5
L33V, P40A, E90Q	-170.8	31	-23	6	5

Table S4. **Computed interaction energies of the homotypic interface in simulations of trimeric L9-rsCSP complexes.** A T-test (two-tailed) was used to compare the predicted effect of listed mutations on the stability of the homotypic interface relative to L9 in a random subset of frames from each simulation. The table lists the average value across all the frames from the simulation, which corresponds well to the random subsets. *p<<0.0001; p values for non-significant comparisons: R31S: 0.17; L33V/P40A/E90Q: 0.24; L9k/F10H: 0.05. Source data for Table S4 are provided as a Source Data file.

Table S5. Free Fv (apo) aggregated simulation times.

L9 Variant	Aggregated simulation time (μs)
L9 WT	21.6
F28S	18.5
F28S, H70D	19.3
H70D	23.4
R31S	15.9
E68G	19.1
R31S, E68G, H70D	23.9
F28S, R31S, E68G, H70E	25.8

Supplementary References

1 Punjani, A. & Fleet, D. J. 3D variability analysis: Resolving continuous flexibility and discrete heterogeneity from single particle cryo-EM. *J Struct Biol* **213**, 107702, doi:10.1016/j.jsb.2021.107702 (2021).

2 Pettersen, E. F. *et al.* UCSF Chimera--a visualization system for exploratory research and analysis. *J Comput Chem* **25**, 1605-1612, doi:10.1002/jcc.20084 (2004).

3 Emsley, P., Lohkamp, B., Scott, W. G. & Cowtan, K. Features and development of Coot. *Acta Crystallogr D Biol Crystallogr* **66**, 486-501, doi:10.1107/S0907444910007493 (2010).

4 Afonine, P. V. *et al.* Real-space refinement in PHENIX for cryo-EM and crystallography. *Acta Crystallogr D Struct Biol* **74**, 531-544, doi:10.1107/S2059798318006551 (2018).

5 Wang, L. T. *et al.* A Potent Anti-Malarial Human Monoclonal Antibody Targets Circumsporozoite Protein Minor Repeats and Neutralizes Sporozoites in the Liver. *Immunity* **53**, 733-744 e738, doi:10.1016/j.immuni.2020.08.014 (2020).

6 Wang, L. T. *et al.* The light chain of the L9 antibody is critical for binding circumsporozoite protein minor repeats and preventing malaria. *Cell Rep* **38**, 110367, doi:10.1016/j.celrep.2022.110367 (2022).

7 Montiel-Garcia, D., Rojas-Labra, O., Santoyo-Rivera, N. & Reddy, V. S. Epitope-Analyzer: A structure-based webtool to analyze broadly neutralizing epitopes. *J Struct Biol* **214**, 107839, doi:10.1016/j.jsb.2022.107839 (2022).
