# Supplementary material for: Structural basis of epitope selectivity and potent protection from malaria by PfCSP antibody L9
Source: Nat Commun. 2023 May 17;14:2815. doi: 10.1038/s41467-023-38509-2 (PMC10192352; doi:10.1038/s41467-023-38509-2)
Supplement: Supplementary file 3 — Description of Additional Supplementary Files [file 41467_2023_38509_MOESM3_ESM.pdf]

Description of Additional Supplementary Files

File Name: Supplementary Movie 1

Description: Snapshots of the MD simulations of the F28S mutant, showing the destabilization of the homotypic interface as a consequence of the mutation. The interacting residues in the homotypic interface are shown as sticks and the interactions are depicted with black dashed lines.
